# CREB1 directly activates the transcription of ribonucleotide reductase small subunit M2 and promotes the aggressiveness of human colorectal cancer

**DOI:** 10.18632/oncotarget.12938

**Published:** 2016-10-27

**Authors:** Zejun Fang, Aifen Lin, Jiaoe Chen, Xiaomin Zhang, Hong Liu, Hongzhang Li, Yanyan Hu, Xia Zhang, Jiangang Zhang, Lanlan Qiu, Lingming Mei, Jimin Shao, Xiang Chen

**Affiliations:** ^1^ Central Laboratory, Sanmen People's Hospital of Zhejiang, Sanmen, Zhejiang, 317100, China; ^2^ Human Tissue Bank, Taizhou Hospital of Zhejiang Province, Wenzhou Medical University, Linhai, Zhejiang, 317000, China; ^3^ Zhejiang Normal University - Jinhua People's Hospital Joint Center for Biomedical Research, Jinhua, Zhejiang, 321004, China; ^4^ Department of Pathology and Pathophysiology, Zhejiang University School of Medicine, Hangzhou, Zhejiang, 310058, China

**Keywords:** CREB1, ribonucleotide reductase small subunit M2, aggressiveness, colorectal cancer

## Abstract

As the small subunit of Ribonucleotide reductase (RR), RRM2 displays a very important role in various critical cellular processes such as cell proliferation, DNA repair, and senescence, etc. Importantly, RRM2 functions like a tumor driver in most types of cancer but little is known about the regulatory mechanism of RRM2 in cancer development. In this study, we found that the cAMP responsive element binding protein 1 (CREB1) acted as a transcription factor of RRM2 gene in human colorectal cancer (CRC). CREB1 directly bound to the promoter of RRM2 gene and induced its transcriptional activation. Knockdown of CREB1 decreased the expression of RRM2 at both mRNA and protein levels. Moreover, knockdown of RRM2 attenuated CREB1-induced aggressive phenotypes of CRC cells *in vitro* and *in vivo*. Analysis of the data from TCGA database and clinical CRC specimens with immunohistochemical staining also demonstrated a strong correlation between the co-expression of CREB1 and RRM2. Decreased disease survivals were observed in CRC patients with high expression levels of CREB1 or RRM2. Our results indicate CREB1 as a critical transcription factor of RRM2 which promotes tumor aggressiveness, and imply a significant correlation between CREB1 and RRM2 in CRC specimens. These may provide the possibility that CREB1 and RRM2 could be used as biomarkers or targets for CRC diagnosis and treatment.

## INTRODUCTION

As an enzyme of central importance in DNA synthesis, Ribonucleotide reductase (RR) plays a crucial role in various biological processes by catalyzing the de novo conversion of ribonucleoside diphosphates to deoxyribonucleoside diphosphates [1]. The RR holoenzyme is composed of two identical large subunits (RRM1) and two identical small subunits (RRM2 or RRM2B). Previous studies have investigated the structures and functions of the three RR subunits [2]. RRM1–RRM2 holoenzyme is necessary for S-phase DNA replication and repair in proliferating cells, whereas RRM1–RRM2B provides dNTPs for DNA repair in quiescent cells as well as mtDNA replication and repair [3–5]. Due to that RRM1 remains constant throughout the cell cycle owing to its long halflife, the activity of RR is modulated by levels of the small subunits (RRM2 or RRM2B), expressions of which are tightly regulated during the cell cycle [6–8]. As a major mechanism for RR regulation, transcriptional regulation is applied to control this pivotal enzyme under different physiological conditions [9]. Several transcription factors such as E2F, are responsible for S phase induced transcription of RRM2 while p53 is known as a novel activator of RRM2B in response to DNA damage [10–12].

cAMP responsive element binding protein 1 (CREB1) which belongs to the basic leucine zipper (bZIP) family is a well characterized transcription factor that mediates the transduction between the upstream signal and downstream gene transcription [13]. As a transcriptional activator, CREB1 binds to cyclic-AMP response element (CREs) sequences located at the promoters of target genes and induce their transcription [14]. Abnormal expression of CREB1 was observed in many cancers including acute myeloid leukemia, breast cancer, non-small cell lung carcinoma and renal cancer [15–17]. Like a oncogene, CREB1 is involved in a number of tumor cellular processes such as proliferation, invasion, and metastasis [18, 19]. Recent researches reported that tumor associated genes including Bcl-2, c-fos, and tumor necrosis factor-α (TNF-α) were regulated by CREB1 [20, 21].

In the present study, we discovered that CREB1 could directly bind to the promoter of RRM2 and induce its transcription in CRC cells. Moreover, *in vitro* and *in vivo* experiments confirmed that CREB1-RRM2 pathway promoted the proliferation, migration, and invasion of CRC cells. Consistent with the study in CRC cells, a significant correlation between CREB1 and RRM2 was found by analyzing the data from TCGA database and clinical CRC specimens. Altogether, these results demonstrated that the relationship between CREB1 and RRM2 has potential for future clinical applications in diagnosis and treatment of CRC.

## RESULTS

### CREB1 increases RRM2 expression in CRC cells

**Figure 1:**
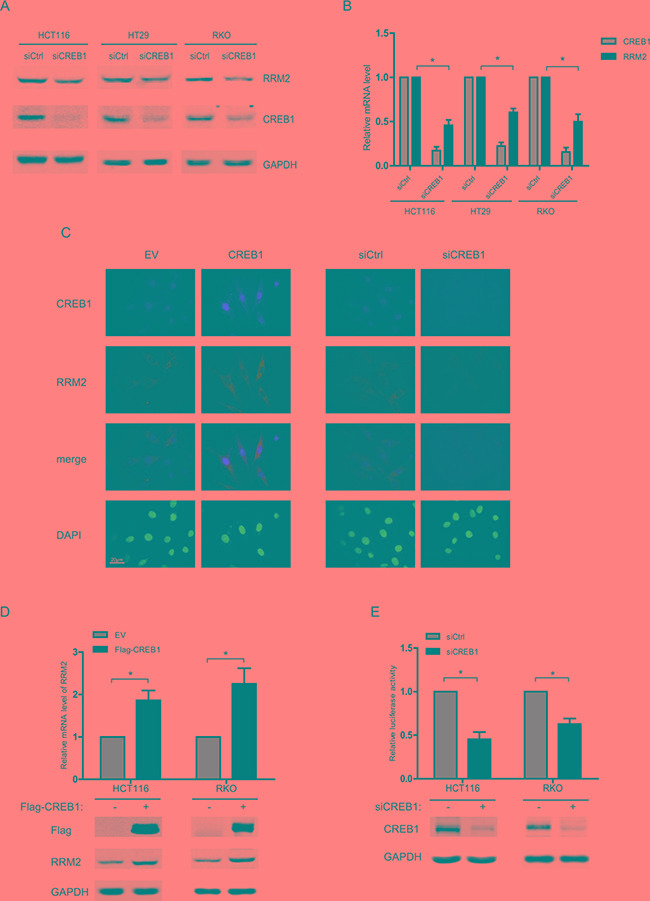
CREB1 increases RRM2 expression in CRC cells **A.** HCT116, HT29, and RKO cells were transfected with either control siRNA or CREB1 siRNA for 48 h, and harvested for Western blot analysis with antibodies anti-CREB1, anti- RRM2, and anti-GAPDH (as loading control). **B.** HCT116, HT29, and RKO cells were transfected with control or CREB1 siRNA for 48 h. The mRNA levels were analyzed by qPCR (normalized by actin). *P <0.05. **C.** HCT116 cells were seeded onto the coverslips in culture dishes. Cells were transfected with indicated siRNA or expression plasmids for 48 h, fixed and then immunoflourescence assay was performed. DAPI served as nuclear marker. **D.** HCT116 or RKO cells were transfected with empty vector (EV) or CREB1 expression plasmid, and then harvested for Western blots and RNA expression analysis 48 h later. *P <0.05. **E.** HCT116 or RKO cells were transfected with control siRNA or CREB1 siRNA as well as RRM2 reporter (−2465/+23) and an internal control reporter pRL-TK for 48 h. *P <0.05.

To verify CREB1 as a regulator of RRM2, CREB1-targeting siRNA was introduced into several CRC cell lines and the expression levels of RRM2 were examined. In all three cell lines, silencing CREB1 with siRNA resulted in the down-regulation of RRM2 protein (Figure 1A). Further, to test whether the correlation extended to the mRNA level, three CRC cell lines were transfected with CREB1 siRNA and the mRNA levels of RRM2 were measured. Knockdown of CREB1 significantly decreased RRM2 at mRNA level (Figure 1B), and immunofluorescence assays also indicated the reduction of RRM2 after CREB1 depletion (Figure 1C). These results suggested that the expression of RRM2 is activated by CREB1 in CRC cells. Consistently, through immunofluorescence assay, western blot, and qRT-PCR, overexpression of CREB1 was observed to promote the expression of RRM2 at both mRNA and protein levels in HCT116 and RKO cell lines (Figure 1C and 1D). To further understand the underlying mechanisms, the transcriptional activity of RRM2 promoter was determined using reporter gene assay in HCT116 and RKO cell lines with CREB1 knockdown. The results showed a significant reduction of RRM2 promoter activity after knockdown of CREB1 in HCT116 and RKO cells (Figure 1E), which revealed that CREB1 may induce RRM2 expression mainly by transcriptional activation.

### CREB1 directly binds to RRM2 promoter and induces its transcription

**Figure 2:**
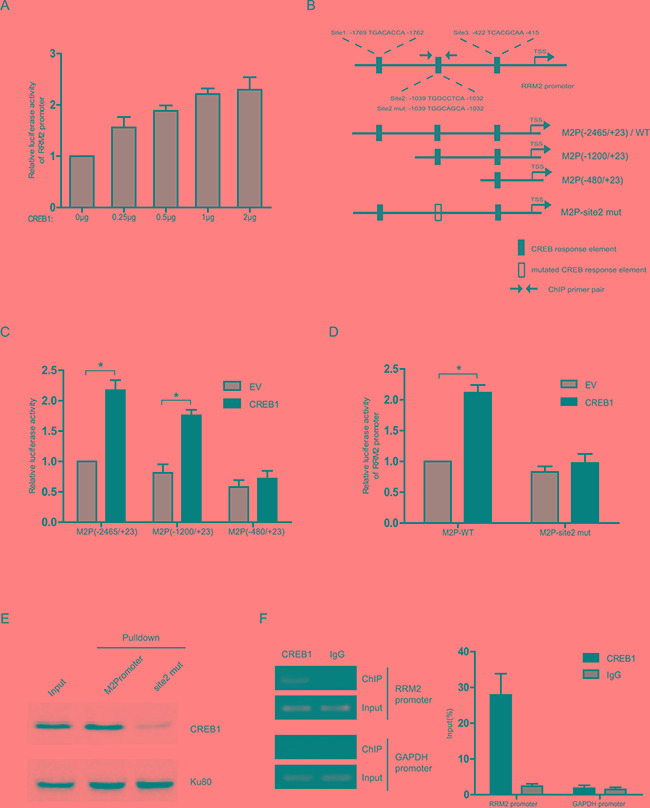
CREB1 directly binds to RRM2 promoter and induces its transcription **A.** Relative luciferase activity in HCT116 cells co-transfected with different amounts of E2F1 expression plasmid, RRM2 promoter reporter (−2465/+23), and an internal control reporter pRL-TK. **B.** The graph shows three putative cyclic-AMP response elements (CREs) on the RRM2 promoter, where CREB1 could potentially bind to and activate RRM2 transcription. **C** and **D.** Transcription activity in response to CREB1 overepression for 48 h was measured by luciferase assay in HCT116 cells with a series of truncated or mutated RRM2 promoter reporter (internal control, pRL-TK). *P <0.05. **E.** In HCT116 cells, the binding ability of CREB1 to the biotin-labeled RRM2 promoter probe (−2465/+23) was analyzed by DNA pull-down assay. Ku80 served as a control. **F.** Chromatin prepared from HCT116 cells was immunoprecipitated with the indicated antibodies; PCR and qPCR were performed on immunoprecipitated DNAs or soluble chromatin using specific primer pairs for the RRM2 promoter and GAPDH promoter (as negative control).

Considering CREB1 as an important transcription factor in various cellular processes, we hypothesized that CREB1 could activate the transcription of RRM2 directly. Ectopic expression of CREB1 increased the transcription activity of RRM2 promoter (Figure 2A). The promoter sequence of RRM2 gene was analyzed by using the programs JASPAR and TFsearch. Three potential cyclic-AMP response elements (CREs) were predicted within the 2-kb upstream region of the promoter (Figure 2B). Serial truncated constructs of RRM2 promoter were examined by luciferase reporter assays to identify the transcriptional regulatory region responsive to CREB1. The results indicated that the RRM2 promoter without the region between -1200 and -480 lost the ability to be activated by CREB1 (Figure 2C). Further analysis showed that this region contained a putative CRE (site2). Mutation in CRE-site2 markedly reduced the reporter activity activated by CREB1 (Figure 2D). For examining whether CREB1 directly binds to the promoter of RRM2, *in vitro* DNA pull-down assay was carried out with nuclear protein of HCT116 cells. DNA pull-down experiments confirmed the inability of DNA probe containing a mutated CRE-site2 to bind CREB1 compared with the wild-type in HCT116 (Figure 2E). Next, chromosome-immunoprecipitation (ChIP) assays were performed to determine that CREB1 binds to RRM2 promoter under physical condition. The evidence showed that CREB1 directly bound to the RRM2 promoter region around CRE-site2 (−1039/−1032) (Figure 2F). In summary, these data demonstrated that CREB1 specifically binds to RRM2 promoter and activates its transcription in CRC cells.

### RRM2 mediates CREB1-induced proliferation, migration, and invasion of RKO cells

**Figure 3:**
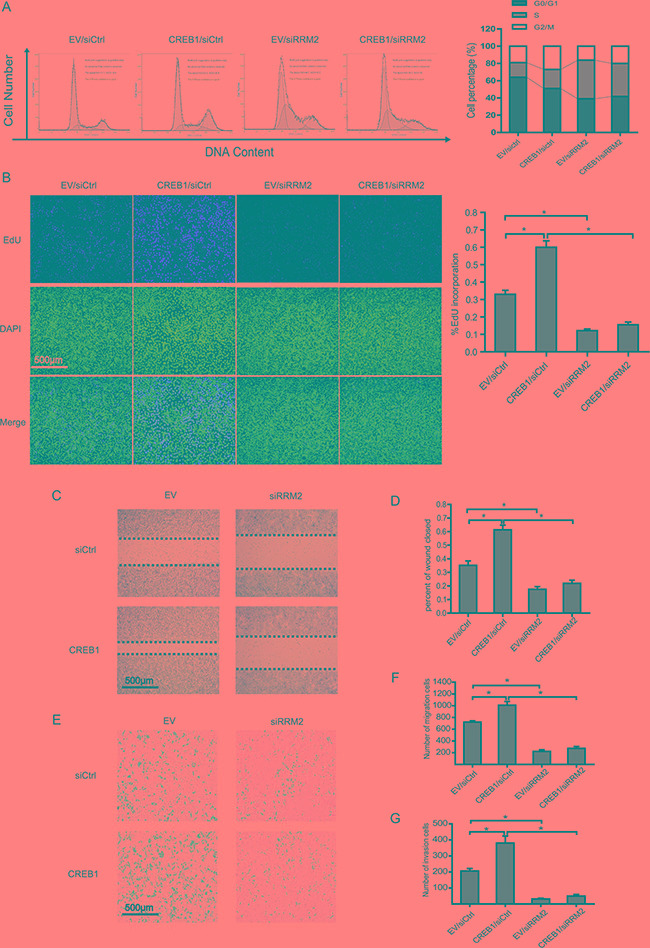
RRM2 mediates CREB1 induced proliferation, migration, and invasion of RKO cells **A.** Cell cycle profiles of RKO cells transfected with the indicated siRNA or plasmids by flow cytometry. DNA content was analyzed using propidium iodide staining and flow cytometry analysis. *P<0.05 **B.** DNA synthesis was measured by EdU incorporation assays in RKO cells after the indicated transfection. *P<0.05. **C-D.** Left panels: images from scratch assays with RKO cells transfected with indicated siRNAs and expression plasmids. Right panels: percentage wound closure 48 h after the indicated transfection. *P<0.05. **E-F.** Left panels: representative images of RKO cells migration. Right panels: numbers of migratory cells transfected with the indicated siRNAs and expression plasmids for 48 h. *P<0.05. **G.** Numbers of invasive cells transfected with the indicated siRNAs and expression plasmids for 48 h. *P<0.05.

To investigate the consequence of CREB1 activated RRM2 expression, RKO cells were transfected with CREB1 overexpression plasmids and siRNA targeting RRM2. Flow cytometry and EdU incorporation assays were implemented to examine how RRM2 affected the proliferation of CRC cells, which was induced by CREB1. It was shown that overexpression of CREB1 boosted cell cycle progression and DNA synthesis (Figure 3A and 3B). However, knockdown of RRM2 not only impaired the effects induced by overexpression of CREB1 but also led to S phase arrest (Figure 3A and 3B). Consistently, the significance of CREB1-RRM2 pathway on cell proliferation was also confirmed in HCT116 cells through knocking down CREB1 coupled with overexpression of RRM2 (Supplementary Figure S1A and S1B). Our previous work has reported that RRM2 contributes to tumor invasion and metastasis in CRC [22]. The roles of CREB1 and RRM2 in migration and invasion of CRC cells were investigated by wound healing and cell invasion assays. The results suggested that abrogation of RRM2 by siRNA in RKO cells reduced its migration and invasion while overexpression of CREB1 stimulated the aggressiveness of RKO cells (Figure 3C–3G). Notably, CREB1 overexpression was not able to restore the incapability of migration and invasion in RKO cells with knockdown of RRM2 (Figure 3C–3G). However, overexpression of RRM2 partially recovered the malignant phenotypes of HCT116 cells, which was attenuated by knockdown of CREB1 (Figure S1C-G). Therefore, these results indicated an important role of CREB1-RRM2 pathway in CRC development.

### Deprivation of RRM2 inhibits CREB1-stimulated CRC tumourigenesis in nude mice

**Figure 4:**
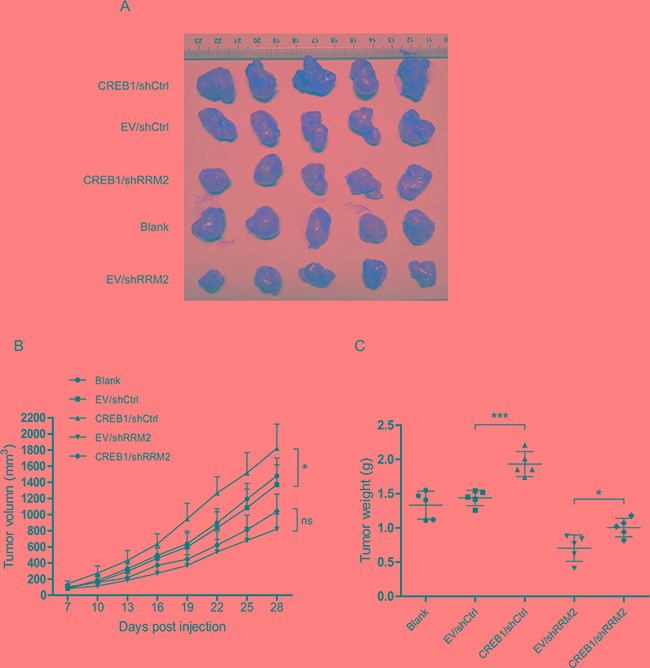
Deprivation of RRM2 inhibits CREB1-stimulated CRC tumourigenesis in nude mice **A.** Images of tumors formed by RKO cells with indicated treatment. **B.** The growth curves of the tumors formed by the indicated RKO cells. The data are presented as the mean ± SD (N = 5 mice per group). *P<0.05. **C.** Weight differences in tumors formed by the indicated RKO cells. The data are presented as the mean ± SD (N = 5 mice per group). *P<0.05, ***P<0.001.

To further determine the role of CREB1-RRM2 pathway in tumorigenesis *in vivo*, RKO cells with stable knockdown of RRM2 and overexpression of CREB1 were subcutaneously injected into nude mice. Tumor formation was evaluated 28 days after injection. Results demonstrated that overexpression of CREB1 resulted in a significant promotion of RKO xenograft growth in nude mice while the repression of RRM2 significantly reduced the volumes and weights of the xenografts (Figure 4A–4C). Importantly, abrogation of RRM2 by shRNA apparently damaged the promotive effect of CREB1 overexpression on RKO xenograft growth (Figure 4A–4C). Collectively, our findings showed that RRM2 displays a critical role in CREB1-induced CRC tumorigenesis *in vivo*.

### Abnormal high expressions of CREB1 and RRM2 indicates a poor prognosis in CRC patients

**Figure 5:**
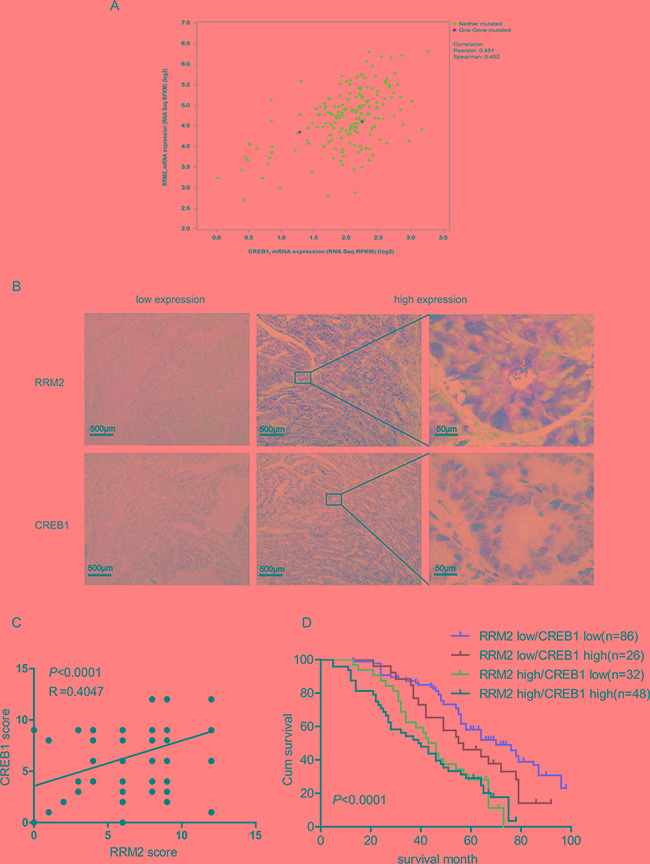
Abnormal high expressions of CREB1 and RRM2 indicate a poor prognosis in CRC patients **A.** Analysis of the TCGA colorectal adenocarcinoma database(TCGA, Nature 2012) using cBioPortal showing the correlation between CREB1 and RRM2 mRNA levels **B.** Representative images of CREB1 and RRM2 immunostaining in human colorectal cancer tissues. **C.** The correlation of concurrent immunostaining scores of RRM2 and CREB1 in CRC tissues. **D.** The survival rate plot of indicated expression groups in CRC. The cumulative 5-year survival rate of the patients with RRM2 and CREB1 double-low expressions was 58.14%, while that of the patients with RRM2-high and CREB1-high expressions was 29.17%. The survival rates between RRM2/CREB1 double-low and double-high expressions groups were significantly different (*P* <0.0001).

The data from TCGA database (Colorectal Adenocarcinoma: TCGA, Nature 2012 [23]) was analyzed to determine the correlation between CREB1 and RRM2 at mRNA level in clinical CRC specimens by the cBioPortal platform. A significant relationship between CREB1 and RRM2 at mRNA level were observed, as revealed on the log2-transformed chart (Figure 5A). With a threshold of >0.3 or <−0.3 in either Pearson or Spearman score, CREB1 was positively correlated with RRM2. Moreover, immunohistochemical staining was performed to evaluate the relevance of CREB1 and RRM2 at protein level in 192 CRC patients (Figure 5B). And further analysis showed that the RRM2 and CREB1 staining was positively correlated with lymph node metastasis, distant metastasis and advanced TNM stages (p<0.05) (Table 1). Moreover, the RRM2 expression levels paralleled the changes of CREB1 in the CRC cases as shown by immunohistochemical analyses (p<0.05) (Figure 5C and Supplementary Table S1). Besides, 5-year overall survival (OS) rate of the patients with CREB1 and RRM2 double-high expressions was significantly lower than that of the patients with double-low expressions of CREB1 and RRM2 (29.17% vs 58.14%, p<0.0001). Of note is that patients with low levels of RRM2 have a better prognosis than RRM2-high expression patients regardless of the CREB1 expression status (Figure 5D). Above results verified a positive correlationship between CREB1 and RRM2 in clinical CRC specimens, which is involved in cancer progression and indicates a poor prognosis for CRC patients.

**Table 1:** Correlation of the expression of RRM2 and CREB1 with clinicopathological features in CRC

		Cases	RRM2 expression			CREB1 expression		
			low	high	*P*	low	high	*P*
		192	112	80		118	74	
Tumor location					0.7867			0.6207
	Colon	80	44	36		45	35	
	Rectum	112	68	44		73	39	
Gender					0.2450			0.6537
	male	106	60	46		64	42	
	female	86	52	34		54	32	
Age					0.3026			0.6039
	≤65	83	52	31		45	38	
	>65	109	60	49		73	36	
Tumor size					0.6394			0.5050
	<5cm	87	52	35		55	32	
	≥5cm	105	60	45		63	42	
LNM					0.0172*			0.0276*
	N0	107	70	37		72	35	
	N1/2	85	42	43		46	39	
TNM					0.0104*			0.0022*
	I	28	20	8		18	10	
	II	71	46	25		51	20	
	III	74	42	32		44	30	
	IV	19	4	15		5	14	
Distant metastasis					0.0018*			0.0017*
	M0	173	106	67		106	67	
	M1	19	4	15		5	14	

LNM: lymph node metastasis;

* *P*<0.05

## DISCUSSION

Abnormal expression of CREB1 has been reported in a number of human cancers including solid tumors and hematological malignancy. In acute myeloid leukemia (AML), CREB1 plays a critical role in boosting cell proliferation by regulating RFC3 expression and then promoting the G1/S progression [24]. Besides, high expression of CREB1 is associated with metastasis in gastric and breast cancer [25, 26], while knockdown of CREB1 could inhibit liver cancer cell migration [27]. Consistently, CREB1 has also been identified to be highly expressed in glioma tissues and promote cell growth by activating the expression of oncogenic microRNA-23a [28], and plays an important role in the tumorigenesis of renal cancer by loss of tumor suppressive miR-10b-5p and miR-363-3p [29]. However, CREB1 functions like a tumor suppressor in other cancer types. Some evidences showed that CREB1 inhibited cell proliferation in glioblastoma [30]. In the urinary bladder urothelial carcinoma, CREB1 also plays a tumor suppressor role by transactivating epithelial membrane protein 2 (EMP2)[31]. In the present study, we discovered that expression level of RRM2 parallels with that of CREB1 and RRM2 is a target gene of CREB1 in CRC cells. Knockdown of either CREB1 or RRM2 suppresses cell proliferation, migration, and invasion *in vitro* and *in vivo*. Interestingly, ectopic expression of CREB1 could not recover the impaired malignant phenotypes of CRC cells with knockdown of RRM2 while overexpression of RRM2 could partially rescue the aggressive capacities which are attenuated by silencing CREB1 in CRC cells. These data suggested that RRM2 behaves as a critical effector which promotes CREB1 induced aggressiveness of CRC cells. In addition to increasing RRM2 expression, CREB1 also performs a role in inhibiting cell apoptosis by activating PKD1/CREB/Bcl-2 pathway in CRC [32]. Coincidently, RRM2 could also increase Bcl-2 protein stability in Head and Neck and Lung Cancers [33]. So the CREB1-RRM2 pathway may have a more significant role in resisting apoptosis in CRC cells, which needs further investigations. So we hypothesized that CREB1 contributes to CRC development partially by promoting RRM2 expression.

RRM2 was identified as a tumor promoter in most cancer types and its expression was evidently higher in malignant carcinoma. However, the abnormal expression level of RRM2 is a consequence of dysregulation in most cancer cells. Though some regulatory factors have been introduced to regulate the expression of RRM2, the underlying mechanisms for controlling the crucial enzyme remain unclear in different cancer types. In our previous study, E2F1 was found acting as a tumor driver by activating the transcription of RRM2 in CRC [22]. Dysregulation of E2F1 in CRC is positively related with the abnormal expression of pRb, an important regulator of E2F1 [34, 35]. In addition, upregulation of NF-Y transactivates RRM2 transcription, which played a pivotal role in the Gem resistant KB cells [36]. Whereas, in this study, CREB1 was described as another important transcription factor of RRM2, which indicated that multi-factors or pathways are involved in transcriptional regulation of the pivotal gene. In clinical CRC specimens, the correlation between CREB1 and RRM2 was also verified both at mRNA and protein levels.

To further understand the mechanisms underlying ectopic expression of CREB1 in CRC, we analyzed the previous reports on CREB1 promoter. Interestingly, an E2F1 binding site was found located at the promoter of CREB1 [24]. Therefore, we examined the relationship between E2F1 and CREB1 in CRC cells. As shown in Supplementary Figure S2, overexpression of E2F1 evidently elevates the mRNA and protein levels of both CREB1 and RRM2 in CRC cells (Supplementary Figure S2A-S2C). Consistently, a reversed alteration of CREB1 and RRM2 expression was observed in CRC cells with knockdown of E2F1 (Supplementary Figure S2D-S2F). So, abnormal expression of E2F1 in CRC, which has been reported in our previous studies, may contribute to induce the transcription of CREB1 and consequently result in deregulated RRM2 expression. In summary, except for the direct regulation of RRM2 by E2F1, E2F1-CREB1-RRM2 signal pathway may be also employed in CRC development. Apart from increased protein level, the roles of CREB1 also depend on its phosphorylation, which is up-regulated by cAMP-PKA pathway. Aberrant activation of cAMP-PKA-CREB1 signal pathway was observed in a number of cancer types [37, 38]. However, considering that the phosphorylation of CREB1 (p-CREB1) is necessary for its transcriptional activity [14], the upstream signal pathways for activating CREB1 are worth being explored in CRC.

As a target gene of CREB1, RRM2, except for providing dNTPs for cell proliferation, cooperates with a number of oncoproteins to promote tumor development. For instance, RRM2 enhances cellular invasiveness by NF-kB-induced MMP-9 activation in pancreatic ductal adenocarcinoma [39]. Besides, some evidences suggest that RRM2 can enhance tumor angiogenesis by decreasing thrombspondin-1 and increasing VEGF production [40]. Moreover, aberrant RRM2 expression could enable cells to overcome senescence or apoptosis, which is a barrier to transformation [41, 42]. In our study, further analyses of clinical specimens from CRC patients showed that the expression level of either RRM2 or CREB1 was positively correlated with TNM stage and distant metastasis, which is consistent with previous studies and the results of experiments implemented in CRC cells. As expected, the patients with high levels of CREB1 and RRM2 have a relative worse prognosis, compared with others.

In conclusions, our study in CRC cells demonstrated that CREB1 directly binds to the promoter of RRM2 and activate its transcription. As a result, the upregulation of RRM2 induced by CREB1 contributes to proliferation, migration, and invasion of CRC cells. Consistently, high expression of either CREB1 or RRM2 is associated with metastasis and indicates an ominous outcome for CRC patients. The present study may shed light on diagnosis and treatment for CRC.

## MATERIALS AND METHODS

### Cell cultures

HCT116, HT29, and RKO were cultured in RPMI 1640 supplemented with 10 % fetal bovine serum (Gibco, Carlsbad, CA) at 37°C in a humidified 5 % CO_2_ atmosphere.

### Transfection and siRNA interference

The Flag-CREB1, Flag-RRM2, and cmyc-E2F1were transfected with X-treme GENE HP DNA Transfection Reagent (Roche Applied Science, Mannheim, Germany) according to the manufacturer's protocol. CREB1 small interfering RNA (siRNA), RRM2 siRNA, E2F1 siRNA, and scrambled siRNA (Santa Cruz Biotechnology, TX, USA) were transfected with Lipofectamine™ RNAiMAX (Invitrogen, NY, USA) according to the manufacturer's instructions.

### Western blot analysis

The whole cell lysate were analyzed with the antibodies mouse anti-human RRM2, rabbit anti-human CREB1, rabbit anti-human E2F1, mouse anti-human Flag, mouse anti-human cmyc, mouse anti-human GAPDH (Santa Cruz Biotechnology), IRDye® 800CW- or IRDye® 680-conjugated secondary antibodies (LI-COR, Lincoln,NE) were used for staining and then detected by an Odyssey® infrared imaging system (LI-COR).

### Immunofluorescence

HCT116 cell layers on glass coverslips were fixed for 15 min using 4% paraformaldehyde, permeabilized for 20 min in PBS containing 0.2% Triton X-100, and then blocked for 2 h with PBS containing 1% BSA and 0.5% goat serum at 37°C. The cells were incubated with primary antibody at 4°C overnight. The antibodies mouse anti-human RRM2, rabbit anti-human CREB1 were purchased from Santa Cruz Biotechnology (Santa Cruz, CA, USA). After rinsing with PBS, and then probed with FITC- or TRITC- conjugated secondary antibodies (Santa Cruz, CA, USA) for 1 h at 37°C. The nuclei were stained with DAPI (Sigma) for 15 min. The slides were mounted and visualized by a fluorescence microscope (AX70, Olympus, Tokyo, Japan). The images are representative of triplicate independent experiments.

### Quantitative real time RT-PCR

Total RNA was prepared using the RNAiso™ Plus reagent (Takara, Otsu, Japan) and reverse-transcribed using a PrimeScript™RT reagent kit (Takara). Quantitative PCR was performed with SYBR Green mix (Takara) according to the manufacturer's instructions. β-Actin was used as loading control.

### Reporter gene assay

The truncated or mutated promoter sequences of the human RRM2 gene were amplified by PCR and inserted into the pGL3-Basic luciferase reporter vector (Promega, Madison, WI). Cells were plated onto 24-well plates the day before transfection. The cells were cotransfected with 0.5 μg of firefly luciferase reporter constructs, 0.02 μg of pRL-SV40 Renilla luciferase reporter plasmids (Promega), and 0.5μg Flag-CREB1 using the X-treme GENE HP DNA Transfection Reagent. The luciferase activity was measured by a dual-luciferase reporter assay system (Promega).

### DNA pull-down assay

The DNA fragment of human RRM2 promoter from -2465 to +23 was amplified by PCR with a 5′-biotin-labled reverse primer. A total of 100μg nuclear proteins extracted from HCT116 cells was incubated with wild-type or mutated RRM2 promoter DNA probe. DNA pull-down assay was conducted according to the previous study [43]. The bound proteins were analyzed by SDS-PAGE and western blot (Ku80 as loading control).

### Chromatin immunoprecipitation

Chromatin was cross-linked using 1% formaldehyde for 10 min and sonicated to obtain DNA fragments of 200-500 bp. After centrifugation, the supernatants were subjected to immunoprecipitation overnight at 4°C with antibodies against CREB1 or normal rabbit IgG. Protein A/G PLUS-Agarose (Santa Cruz) were used to isolate the chromatin-antibody complexes. The crosslinking was reversed and the precipitated DNA fragments were purified and analyzed by PCR using the following primer pair for RRM2 promoter: GGGTCTCACTATGTTGCCC(forward) and CCCAGCACTTTGGGAGGCC (reverse).

### Flow cytometry

1×10^6^ RKO or HCT116 cells were re-suspended and fixed with 70% ethanol at -20°C for at least an hour. Before analysis, cells were re-suspended in PBS containing 100 mg/ml RNaseA (Roche) and 50 m g/ml PI (Sigma) for at least half an hour. The staining cells were analyzed immediately on a FACSCalibur flow cytometer (Becton Dickinson, San Jose, CA, USA) using the CellQuest 3.0 software system.

### EdU incorporation assay

DNA synthesis was assessed using the Cell-Light EdU (5-ethynyl-2′-deoxyuridine) DNA Cell Proliferation Kit (RiboBio Co) according to its instruction. Images of the cells were captured with a fluorescence microscope (Nikon, Tokyo, Japan). ImageJ software (National Institutes of Health, Bethesda, MD, USA) was used to count the fluorescent points.

### Wound healing assay

Cells were seeded in six-well plates. After transfection for 24 h, the monolayer was gently and slowly scratched with a pipette tip across the center of the well. After scratching, the well was gently washed several times with PBS to remove detached cells. The well was replenished with fresh medium without serum, and the cells allowed growing for additional 48 h, when images of the stained monolayer were captured on a microscope. The wound was evaluated using ImageJ.

### Cell invasion assays

Cell invasion were assessed in Boyden chambers with Matrigel according to the manufacturer's protocol (Invitrogen). First, an 8-mm-porosity polycarbonate membrane was covered with 200μL of serum-free medium containing 1×10^5^ cells per well. The plates were then incubated with 10% FBS medium for 48 h at 37°C in a 5% CO_2_ incubator. The invasion cells on the bottom surface of the filter were fixed, stained, and counted using optical microscopy.

### Nude mouse xenograft assay

25 female BALB/c nude mice were injected with RKO cells with stable knockdown of RRM2 or overexpression of CREB1. All of the mice were randomly divided into five groups (n=5 of each group), and each received a subcutaneous injection of a viable cell suspension mixture (5×10^6^) containing a 90% RKO cells with indicated treatment. When the tumors could be palpated, tumor size was measured using calipers every three days. All of the mice were sacrificed on the fifth week after injection, and the individual tumors were weighed.

### Immunohistochemistry

A total of 192 human CRC samples were collected at the Sanmen People's Hospital after informed consent had been given by all patients. The immunohistochemistry was performed using an Envision Detection System (DAKO, Carpinteria, CA, USA) according to the manufacturer's instructions. To estimate the score for each slide, at least 8 individual fields at 200× were chosen, and 100 cancer cells were counted in each field. The antibodies for CREB1 (1:100 dilutions) and RRM2 (1:100 dilutions) IHC staining were commercially available from Santa Cruz Biotechnology. The immunostaining intensity was divided into four grades: 0, negative; 1, weak; 2, moderate; and 3, strong. The proportion of positive-staining cells was divided into five grades: 0, <5%; 1, 6 −25%; 2, 26 −50%; 3, 51–75%; and 4, >75%. The staining results were assessed and confirmed by two independent investigators blinded to the clinical data. The percentage of positivity of the tumor cells and the staining intensities were then multiplied in order to generate the IHC score, and graded as low expression (score 0~6) and high expression (score 7~12). Cases with a discrepancy in scores were discussed to obtain a consensus.

### Statistics

A database was created and transferred to SPSS 22.0 and Graphpad Prism 5.0 for Windows. Statistical data analysis was performed using the two-tailed Student's t-test, chi-squared, one-way ANOVA, and the results are presented as the mean ± SD of three separate experiments. A value of *P* < 0.05 was considered statistically significant. Spearman test was used in analyzing the correlation.

## SUPPLEMENTARY MATERIALS FIGURES AND TABLE


