# Supplementary material for: CREB1 directly activates the transcription of ribonucleotide reductase small subunit M2 and promotes the aggressiveness of human colorectal cancer
Source: Oncotarget. 2016 Oct 27;7(47):78055–68. doi: 10.18632/oncotarget.12938 (PMC5363643; doi:10.18632/oncotarget.12938)
Supplement: Supplementary file 1 [file oncotarget-07-78055-s001.pdf]

CREB1 directly activates the transcription of ribonucleotide reductase small subunit M2 and promotes the aggressiveness of human colorectal cancer

SUPPLEMENTARY FIGURES AND TABLE

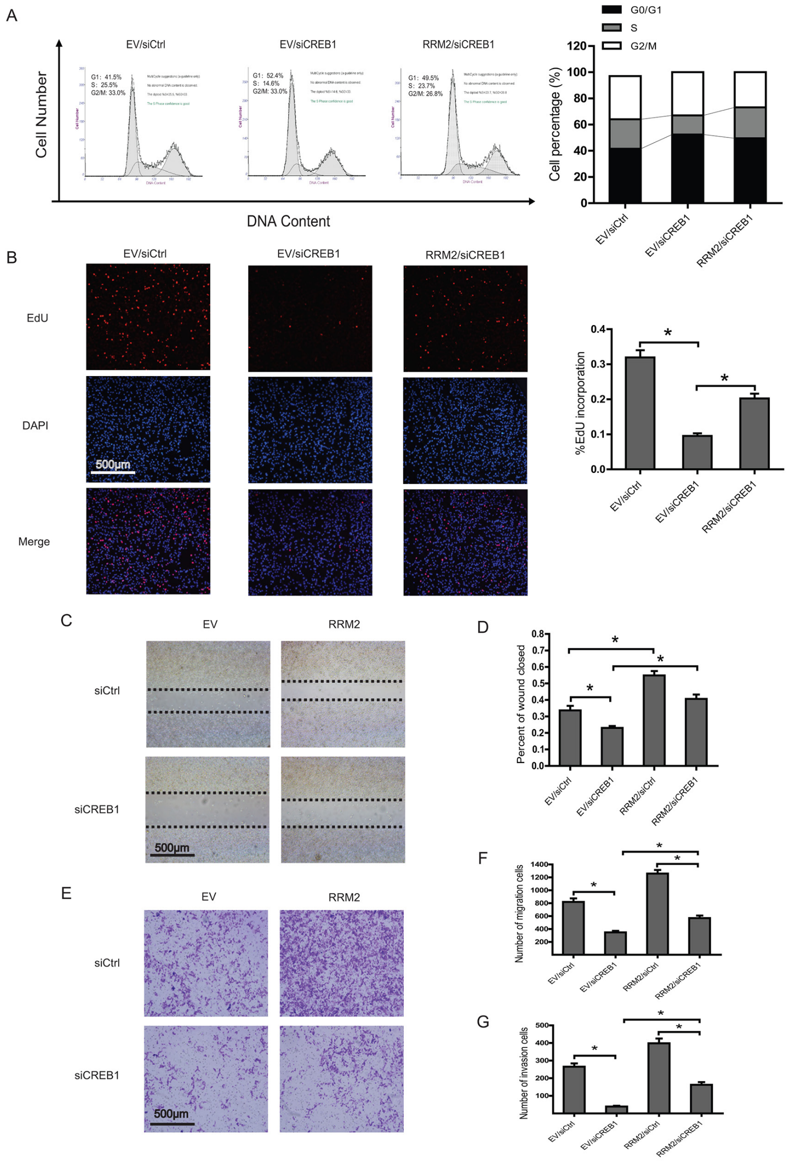

Supplementary Figure S1: The expression of CREB1 and RRM2 promote the proliferation, migration, and invasion of HCT116 cells. A. Cell cycle profiles of HCT116 cells transfected with the indicated siRNA or plasmids by flow cytometry. DNA content was analyzed using propidium iodide staining and flow cytometry analysis. *P<0.05 **B.** DNA synthesis was measured by EdU incorporation assays in HCT116 cells after the indicated transfection. *P<0.05. **C-D.** Left panels: images from scratch assays with HCT116 cells transfected with indicated siRNAs and expression plasmids. Right panels: percentage wound closure 48 h after the indicated transfection. *P<0.05. **E-F.** Left panels: representative images of HCT116 cells migration. Right panels: numbers of migratory cells transfected with the indicated siRNAs and expression plasmids for 48 h. *P<0.05. **G.** Numbers of invasive cells transfected with the indicated siRNAs and expression plasmids for 48 h. *P<0.05.

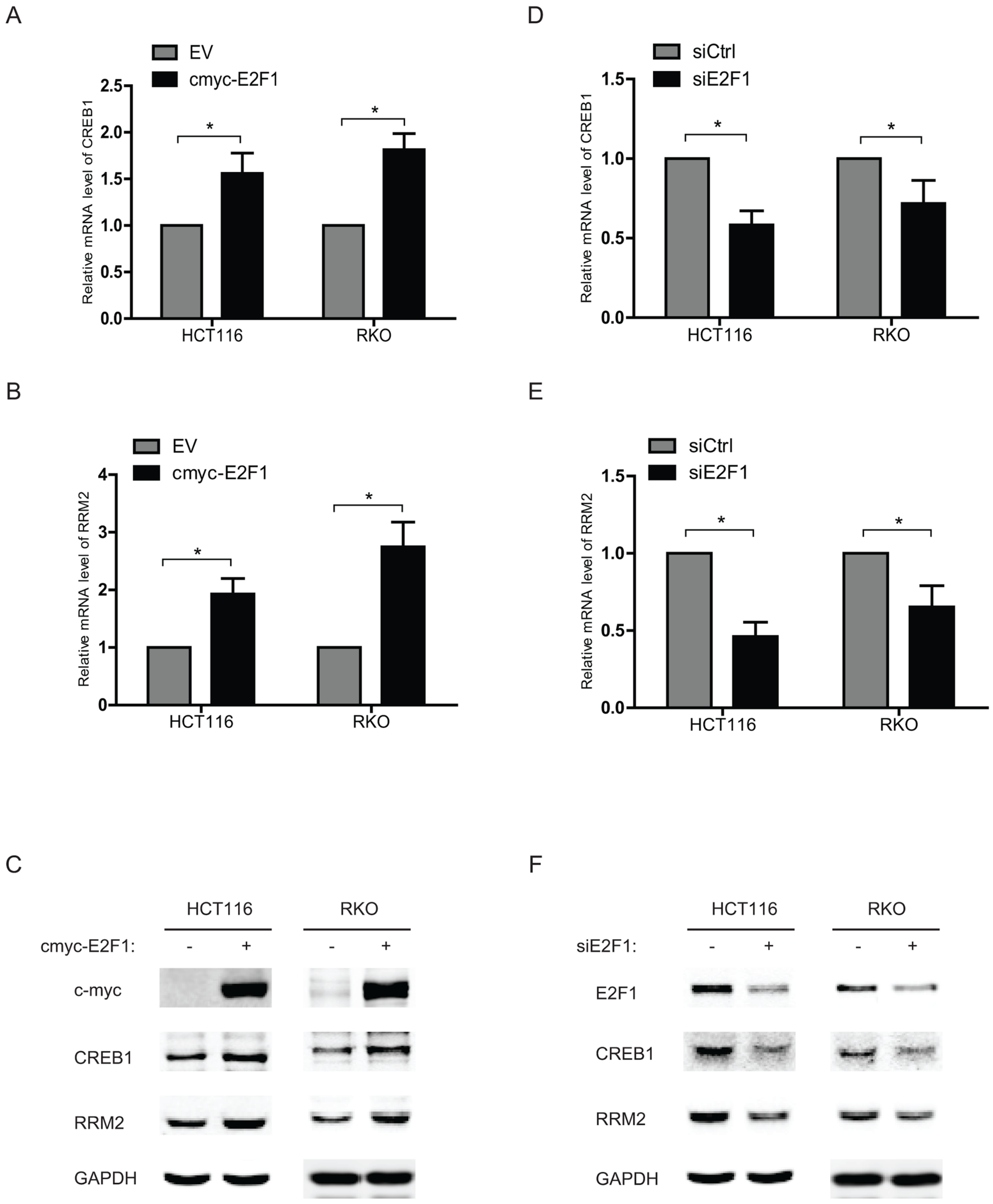

Supplementary Figure S2: The expression of CREB1 is induced by E2F1 in CRC cells. A and **B.** HCT116 and RKO cells were transfected with control or E2F1 overexpression plasmids for 48 h. The mRNA levels of CREB1 (A) and RRM2 (B) were analyzed by qPCR (normalized by actin). *P <0.05. **C.** Same treatments with (A). Cells were harvested for Western blot analysis with antibodies anti-cmyc, anti-CREB1, anti-RRM2, and anti-GAPDH (as loading control). **D-E.** HCT116 and RKO cells were transfected with control or E2F1 siRNA for 48 h. The mRNA levels of CREB1 (D) and RRM2 (E) were analyzed by qPCR (normalized by actin). *P <0.05. **F.** Same treatments with (D). Cells were harvested for Western blot analysis with antibodies anti-E2F1, anti-CREB1, anti-RRM2, and anti-GAPDH (as loading control).

Supplementary Table S1: Relationship between RRM2 and CREB1 expression in CRC

		**RRM2**		**r**	***P***
		**low**	**high**		
CREB1	low	86	32	0.4253	<0.0001
	high	26	48		
